# Pharmacological potential of *Bidens pilosa* L. and determination of bioactive compounds using UHPLC-QqQ_LIT_-MS/MS and GC/MS

**DOI:** 10.1186/s12906-017-2000-0

**Published:** 2017-11-16

**Authors:** Garima Singh, Ajit Kumar Passsari, Pratibha Singh, Vincent Vineeth Leo, Sarathbabu Subbarayan, Brijesh Kumar, Bhim Pratap Singh, Hauzel lalhlenmawia, Nachimuthu Senthil Kumar

**Affiliations:** 1 Department of Biotechnology, Mizoram University, Aizawl, Mizoram 796004 India; 2 SAIF, CSIR-Central Drug Research Institute (CSIR-CDRI), Lucknow, 226012 India; 3 Department of Pharmacy, Regional Institute of Paramedical and Nursing Sciences, Aizawl, Mizoram 796017 India

**Keywords:** *Bidens pilosa* L., Antioxidant, Antimicrobial, Cytotoxicity, GS-MS analysis, UPLC-ESI-MS/MS

## Abstract

**Background:**

Research of natural products from traditionally used medicinal plants to fight against the human ailments is fetching attention of researchers worldwide. *Bidens pilosa* Linn. var. Radiata (Asteraceae) is well known for its folkloric medicinal use against various diseases from many decades. Mizoram, North East India, has high plant diversity and the use of this plant as herbal medicine is deep rooted in the local tribes. The present study was executed to understand the pharmacological potential of *B. pilosa* leaves extract.

**Methods:**

The antimicrobial potential was determined using agar well diffusion and broth microdilution method against bacterial and yeast pathogens. Cytotoxicity was evaluated using MTT and apoptotic DNA fragmentation assays. Further, the antioxidant ability of the extract was analysed using DPPH and ABTS free radical scavenging assay. Mosquitocidal activity was evaluated against third in-star larvae of *C. quinquefasciatus* using dose response and time response larvicidal bioassay. Additionally, the major phenolic and volatile compounds were determined using UHPLC-QqQ_LIT_-MS/MS and GC/MS respectively.

**Results:**

We found that the extract showed highest antimicrobial activity against *E. coli* (MIC 80 μg/mL and IC_50_ 110.04 μg/mL) and showed significant cytotoxicity against human epidermoid carcinoma (KB-3-1) cells with IC_50_ values of 99.56 μg/mL among the tested cancer cell lines.

The IC_50_ values for scavenging DPPH and ABTS was 80.45 μg/mL and 171.6 μg/mL respectively. The extract also showed the high phenolics (72 μg GAE/mg extract) and flavonoids (123.3 μg Quercetin /mg extract). Lastly, five bioactive and six volatile compounds were detected using UHPLC-QqQ_LIT_-MS/MS and GC-MS respectively which may be responsible for the plant’s bioactivities. An anticancerous compound, Paclitaxel was detected and quantified for the first time from *B. pilosa* leaves extract, which further showed the anticancerous potential of the tested extract.

**Conclusion:**

On the basis of the present investigation, we propose that the leaf extract of *B. pilosa* might be a good candidate for the search of efficient environment friendly natural bioactive agent and pharmaceutically important compounds.

## Background

*Bidens pilosa* Linn. var. *Radiata* (Spanish needles or beggar ticks) from the family Asteraceae is an annual weed widely distributed throughout the tropical and sub-tropical regions of the world [1]. In some part of the world the plant is eaten as food whereas in other countries *B. pilosa* is used in traditional medicines [2]. In Mizoram, it is called as vawkpuithal and is reported to treat various diseases and infections, commonly rheumatism, diarrhoea, ear, eyes and tooth ache problems [3]. Plant has a long ethno-medicinal history for treating malaria, skin infections, stomach and liver disorders. This plant is very well documented as a source of natural antimicrobials [4, 5], anti-inflammatory [6, 7], hepatoprotective [8], and cytotoxic against various cancer cells [9, 10]. Phytochemical screening studies of *B. pilosa* showed the presence of phenylpropanoids, polyacetylenes, polyphenols, triterpenes, saponins and alkaloids [11]. The pharmaceutical property of the plant seems to be associated with the bioactive phytochemical compounds, especially sesquiterpene lactones and polyacetylenes, which inhibit the growth of pathogenic microorganisms and the flavonoids, which are considered as effective anti-inflammatory agents [6, 11, 12]. Phytochemicals and essential oil of *B. pilosa* reported to possess exploitable amount phenolic compounds with free readical scavenging potential [11].

Osmotic stress and autoxidation are the natural phenomenon of human physiology resulted in the overproduction of reactive oxygen species that plays an important pathophysiological role in the development of several human diseases including cancer [13]. Natural antioxidants are stable molecules capable to donate an electron to neutralize these free radicals, but sometimes overwhelmed by excessive stress. Intake of antioxidants counteracts the oxidative damage in the human body, protects DNA, and improves biological antioxidant mechanism by trapping the free radicals [14].

On the other hand, development of drug resistance is becoming serious issue to fight against the diseases [15]. For instance, few bacteria have developed resistance against available antimicrobial agents which has resulted in significant public health problems [16, 17]. Herbal medicine has emerges as a health aid during the last 56 decades and showed the bio prospecting for new plant derived drugs [18, 19]. Previous studies has proved the efficacy of several isolated compounds from *B. pilosa* and suggested the plant as a potential anticancer medicinal plant [10, 20]. The specific polyphenols and flavonoids present in *B. pilosa* were not fully elucidated, although caffeoylquinic acid, luteolin, quercetin and others have been reported so far [21, 22].

Keeping these findings in mind, the present work was designed to assess the in vitro antioxidant, antimicrobial, antitumor and mosquitocidal activities of the *B. pilosa* leave extract. Furthermore, the phenolic, anticancerous and volatile compounds were detected and quantified using UHPLC-QqQ_LIT_-MS/MS and GC MS respectively, which further proves the potentiality of the selected plant to be used in health care system.

## Methods

### Plant collection and extract preparation

Fresh leaves of *B. pilosa* were collected from the Botanical Garden, Mizoram University, Mizoram, India during September 2015 based on traditional uses and identified by Dr. Kalidas Upadhyay, Department of Forestry, Mizoram University. Moreover, the collected plant is also identified by the amplification of internal transcribed spaces (ITS) rRNA gene and the sequence has been submitted in NCBI genebank with the accession number MF440588. A voucher specimen was prepared and kept at the collection of Department of Biotechnology, Mizoram University (MZU/BT/26). The healthy leaves were shade dried at room temperature (30 °C ± 2 °C) for 3 days and grounded to make powder by using a blender. Fifty grams of powder was extracted thrice in 750 ml of methanol for 48 h with occasional stirring. The extract was prepared using rotary evaporator (Buchi, India) at 40 °C under reduced pressure and the obtained crude extract was stored at 4 °C.

### Reagents

2,2- Azinobis-3-ethylbenzothiazoline-6-sulphonic acid disodium salt (ABTS), 2,2-diphenyl-1-picrylhydrazyl (DPPH), Dimethyl Sulphoxide, Sodium acetate trihydrate ACS, Ferric chloride hexahydrate A.R., Ferrous sulphate heptahydrate A.R., Folin ciocalteu’s reagent L.R., Gallic acid monohydrate, L-Ascorbic acid A.R., Acetic acid glacial A.R., Sodium carbonate ACS, Potassium persulphate A.R., were purchased from Hi-media, Mumbai, India. 6-hydroxy-2,5,7,8-tetramethylchromane-2-carboxylic acid (trolox), Aluminium chloride AR, and Quercetin ≥95% (HPLC) solid were purchased from Sigma-Aldrich, USA. Acetonitrile, methanol (LC-MS grade) and formic acid (analytical grade) were purchased from Fluka, Sigma-Aldrich (St. Louis, MO, USA). Ultra pure water was obtained from a Direct-Q 8 UV water purification system (EMD Millipore Corporation, Billerica, MA, USA). All other reagents including solvents were of analytical grade and were procured from Hi-Media, Mumbai, India.

### Phytochemical analysis

#### Total phenolic content (TPC) determination

TPC was determined spectrophotometrically by using Folin-ciocalteu method [23]. Serial dilution of the extract was done in the range of 10–100 mg/mL and gallic acid standard was prepared in the range 10–500 mg/mL. An aliquot of 10 μl of extract was mixed with 90 μl folin reagent (1:10 *v*/v in water) and 100 μl of 15% Na_2_CO_3_ to make the 200 μl volume in a 96 well microplate. The mixture was incubated for 1 h in dark and absorbance was recorded using a UV/Vis microplate spectrophotometer (Multiscan™ GO, Thermo Scientific, MA, USA) at 725 nm. The result was expressed as gallic acid equivalent (GAE) per gram of extract based on the standard curve of gallic acid.

### Determination of total flavonoids

Total flavonoids content of the plant extract was determined by using modified aluminium colorimetric method [24]. 150 μl of methanol extract is mixed with 150 μl of 2% ethanolic AlCl3 and allowed to incubated in dark for 1 h and the absorbance was recorded at 420 nm. The total flavonoids content was expressed as μg quercetin equivalent (QE) per mg of plant extract compared with the standard curve of quercetin.

### Determination of antioxidant potential

#### By using DPPH (2,2-Diphenyl-1-picrylhydrazyl) assay

Free radical scavenging capability of methanolic leaves extract of *B. pilosa* was determined by DPPH assay as described by Brand-Williams et al. [25]. Briefly, plant extract (100 μl) was added at different concentration (10–100 μg/ml) in a 200 μl of freshly prepared DPPH methanolic solution (0.1 mM). Reaction mixture was incubated for 30 min in dark and the absorbance was recorded at 517 nm. Ascorbic acid was used as standard and methanol with DPPH used as blank. Triplicate measurements were taken and the ability to scavenge the DPPH radical was noted by using the given formula: % decolouration = [1-(OD Sample/OD Control] X 100. The concentration that reduced the DPPH colour by 50% was determined as IC_50._

#### By ABTS^+.^ Radical Cation discoloration assay

The ABTS free radical scavenging activity was performed by using the method described by Re et al. [26]. ABTS^+^ Inhibition percentage was measured as described earlier (27). The IC_50_ value was analyzed from the graph plotted as the inhibition percentage against the concentration.

### Antimicrobial assays

#### Sample preparation for antimicrobial assay

10 mg sample of crude methanolic extract of *B. pilosa* leaves was resuspended in dimethyl sulfoxide (DMSO). The final concentration was made to 10 mg/ml, which was Diluted to obtain different concentrations (1.0, 5.0, 7.5 and 10.0 mg/mL) to evaluate the antimicrobial potential against all selected test organisms.

### Test strains

Antimicrobial activity of methanolic leaves extract of *B. pilosa was* checked by the agar well diffusion and broth micro dilution methods. Pathogens used for the study were gram positive bacteria *Staphylococcus aureus* (MTCC-96); *Bacillus subtilis* (MTCC-2097) and *Micrococcus luteus* (MTCC-2070); gram negative bacteria *Escherichia coli* (MTCC-739); *Pseudomonas aeruginosa* (MTCC-2453) and a yeast pathogen *Candida albicans* (MTCC-3017), obtained from microbial type culture collection (MTCC), Chandigarh, India.

#### Antimicrobial assay by using agar well diffusion method

Agar well diffusion assay was used for initial antimicrobial screening [27]. Briefly, the optical densities of the tested organisms were adjusted to match a 0.5 McFarland standard with 10^8^ colony forming unit (cfu) /ml and spreaded on agar plates. A 50 μL of extract at different concentrations was added into the 6 mm wells prepared using the sterilized cork borer. DMSO was served as the negative control and readymade impregnated disc of antibiotic tetracycline (20 μg/disc) as positive control. A clear halo zone around the filled wells showed the antibacterial potential [28]. The experiments were performed in triplicates.

#### Antimicrobial assay by using broth micro dilution method

Minimum Inhibitory Concentration (MIC) of was evaluated using broth micro dilution method on 96-well microtiter plate against all selected test organisms [29]. The bacterial culture suspension was prepared to make the final concentration of 1.0 × 10^4^ CFU/mL (OD = 0.403). Plant extract of different concentrations (1–10 mg/ml) was added in 96-well microtiter plate with bacterial culture suspension. Different concentrations of plant extract were kept as blank, bacterial culture in DMSO was used as negative control, and standard antibiotics i.e. ampicillin was used as positive control. The 96 well plates were incubated for 36 h at 37 °C and the OD was taken as 630 nm. Results were documented as IC_50_ values which indicate 50% reduction of bacterial growth. The IC_50_ values were calculated by using calibration curve drawn by using linear regression.

### Cytotoxicity potential of plant extract

#### Cell lines and cell culture

Three cancer cell lines [Cervical cancer cell (HeLa), Human hepato carcinoma (HepG2) and epidermoid carcinoma (KB-3-1)] were selected and screened against the obtained extract as described earlier [30].

### MTT assay

The cytotoxicity of plant extract was tested against three cancer cell lines using MTT assay [31]. All the selected cell lines were grown with cell density of 10 × 10^−4^ cells/well in 100 μl of medium on 96-well plates and incubated for 24 h at 37 °C in 5% CO_2_ incubator chamber. 5% methanolic plant extracts (1–200 μg/mL) were added to the plates. Cells incubated with 5% methanol were used as blank while untreated cells represented positive control. Experiment was performed in triplicate. After the incubation of 72 h, the culture medium was replaced with 20 μl of MTT in each well and again incubated for 4 h. of incubation. DMSO was added to each well and absorbance was recorded at 570 nm. The percentage of cell viability was calculated as previously described [30].

### DNA fragmentation assay

The selected three cell lines were plated at a density of 1x106cells/well, in a 96 well plate. Cells were treated with the methanolic extract (100 μg/ml) and allowed for 48 h incubation. The DNA fragmentation was carried out as per Sarathbabu et al. [32].

### Determination of phenolic compounds by using UHPLC-QqQ_LIT_-MS/MS

#### Preparation of standard solution

Standard bioactive compounds were prepared in methanol with a final concentration of 1 mg/mL in acetonitrile as mentioned earlier by Singh et al. [30]. Briefly, a mixed standard stock solution (1 mg/mL) of five reference compounds was prepared in methanol. The working standard solutions were prepared by appropriate dilution of the mixed standard solution with acetonitrile to a series of concentration ranges from 0.1–1000 ng/mL. The standard stock and working solutions were stored at −20 °C until use and vortexed for 30 s prior to injection.

### UHPLC-QqQ_LIT_-MS/MS conditions

The UHPLC-QqQLIT-MS/MS analysis was performed by following the protocol of Pandey et al. [33] with minor modifications. The optimized compound dependent Multiple Reaction Monitoring (MRM) parameters of each analyte are presented in Table 1.

**Table 1:** Multiple reaction monitoring (MRM) compound dependent parameters for reference analytes

Peak No.	t_R_ (min)	Analytes	Q1 (Da)	Q3 (Da)	DP ^a^(V)	EP^b^ (V)	CE^c^ (eV)	CXP^d^ (V)	Polarity
1	0.83	Catechin	289.0	203.0	−110	−10	−29	−8	Negative
2	1.50	Kaempferol	285.0	239.0	−95	−5	−39	−15	Negative
3	2.82	Ferulic acid	193.0	134.0	−58	−5	−23	−9	Negative
4	3.15	Gallic acid	169.0	125.0	−59	−8	−21	−10	Negative
5	4.16	Paclitaxel	852.3	525.1	−57	−9	−17	−16	Negative

*DP* ^a^ declustering potential, *EP* ^b^ entrance potential, *CE* ^c^ collision energy, *CXP* ^d^ cell exit potential

### Determination of volatile compounds by using gas chromatography mass spectroscopy (GC/MS)

Bioactive volatile compounds present in the *B. pilosa* methanolic leaves extract was analysed and identified using GC/MS as described by Sen et al. [34] and Rufatto et al. [35] with some minor modifications. Analysis was performed on Perkin Elmer Turbo mass with single quadrapole fitted with PE-5MS column (thickness 0.25 μm, length 30 m, internal diameter 25 mm, composed of 100% Dimethyl polysiloxane), operating in electron ionization (EI) mode in 220 °C at 70 eV. Helium (99.999%) was used as carrier gas at a constant flow of 1 ml/min and 1 μl of the sample was injected at 250 °C (split at the ratio of 1:30; ion-source temperature 280 °C). The oven temperature was started at 75 °C held for 5 min and ramped at 10 °C per min up to 280 °C, ending with a 10 min. Mass spectrometer was run in the electron ionization (EI) mode in 220 °C at 70 eV with a scan range of 10 to 620 m/z. The peaks were analysed and identified the mass by comparing the mass stored in the National Institute of Standards and Technology (NIST, USA) library.

### Mosquitocidal potential

#### Mosquito culture and maintenance

*C. quinquefasciatus* larvae were collected from Mizoram University campus during the month of March–April, 2016. The larvae were grown and maintained as per Lalrotluanga et al. [36].

### Larvicidal bioassay

The larvicidal bioassay was carried out according to WHO standard protocols [37] with slight minor modifications. Five different concentrations (concentrations of 50, 100, 200, 400 and 500 ppm) of methanolic plant extract were prepared with sterilized distilled water. For experimental treatment, 1.0 ml of different concentrations of plant extracts individually dissolved in 249 ml of water with around 25 third instar larvae of *C. quinquefasciatus.* No foods were supplied during the treatment. 1 ml of 5% methanol mixed with 249 ml of dH_2_O was used as control. Mortality and dead larvae was documented after 24 h of post-exposure period. The experiments were performed in triplicates at 27 ± 2 °C with 75–85% relative humidity. Larval susceptibility (LC_50_) in ppm and LT_50_ were calculated by probit analysis as per Lallawmawma et al. [38].

### Statistical analysis

The data obtained as the mean of three replicates was analyzed using Microsoft Excel XP 2007. One way ANOVA was used to determine the significant differences (*P* ≤ 0.05) by using SPSS software version 16.0 (IBM SPSS, USA).

## Results

### Total phenolics and flavonoids contents

**Fig. 1:**
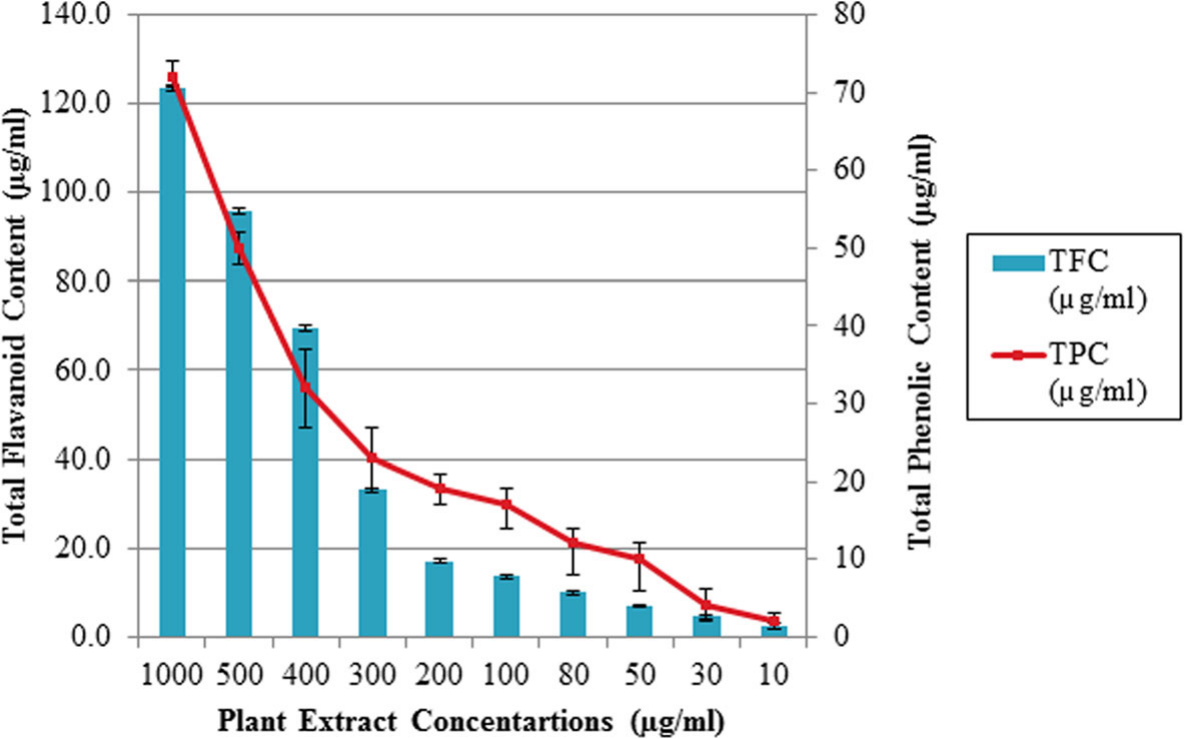
Total phenolic contents and total flavanoids content determined in the leaves extract of *B. pilosa*. Bar represents the means ± SD of triplicate experiments

Total phenolic content (TPC) of *B. pilosa* leaves extract was detected by Folin-Ciocalteu method and result was expressed as mg/GAE equivalent. The extract showed a significant amount of phenolic content of 72 μg of GAE per mg of DW. Total flavonoids content was expressed as milligram of quercetin equivalent and was found to be 123.3 μg Quercetin per mg of DW (Fig 1).

### Antioxidant potential

**Fig. 2:**
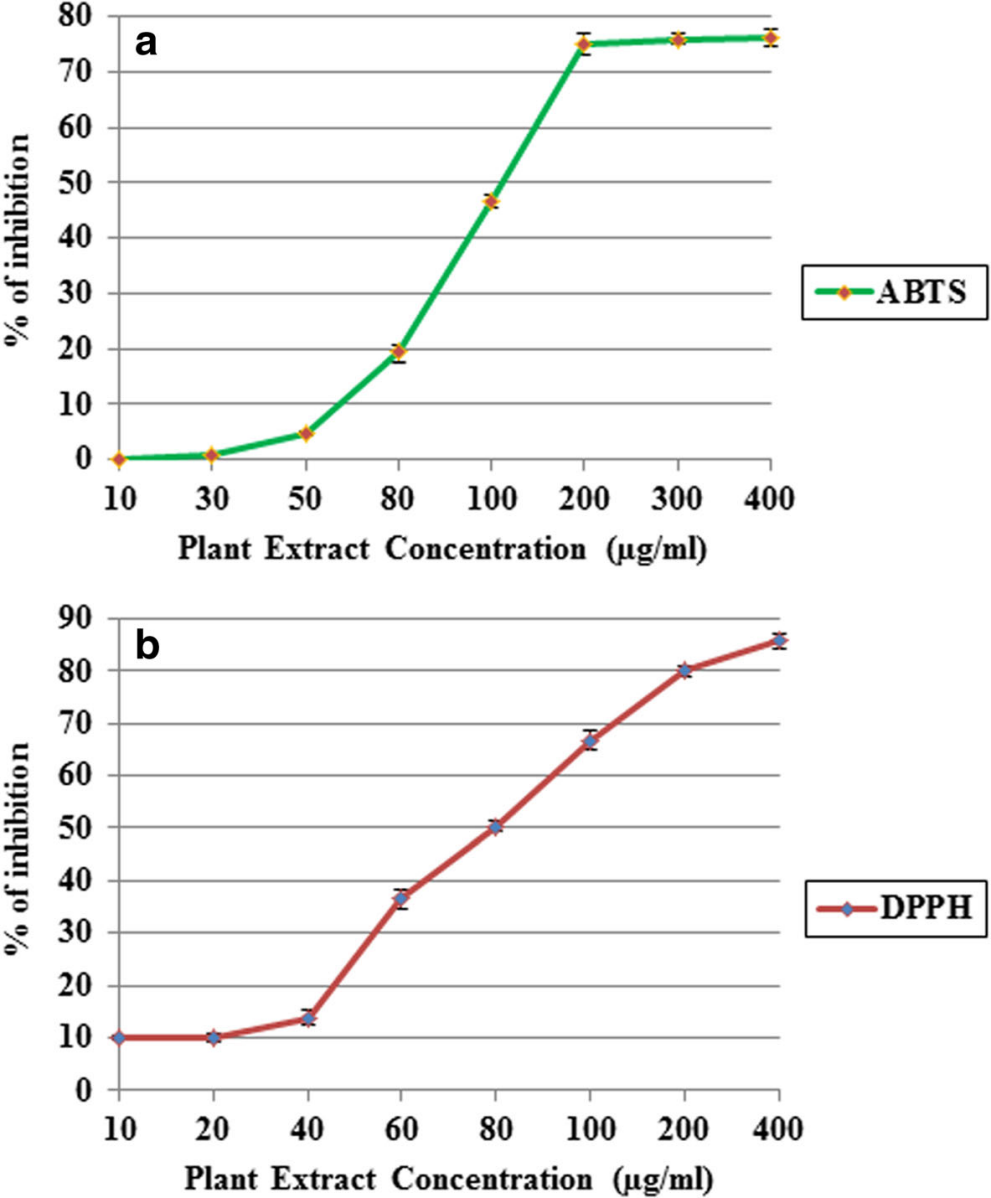
Antioxidant potential of leaves extract of *B. pilosa*. **a** ABTS assay (**b**) DPPH assay

DPPH and ABTS based antioxidant potential of the studied plant extract was estimated by using the IC_50_ values, which is the concentration of the plant extract required for 50% scavenging of DPPH and ABTS radicals in a specific time. The IC_50_ values with respect to DPPH and ABTS scavenging assay were found as 80.45 μg/ml and 171.6 μg/ml respectively, which is a significant antioxidant amount in leaves of *B. pilosa* (Fig 2). Smaller IC_50_ value means higher antioxidant of the plant extract.

### Antimicrobial assay

#### Antimicrobial assay using agar well diffusion method

The results representing the antimicrobial potential of crude methanolic leaves extract of *B. pilosa* is documented in Table 2. The extract showed significant antibacterial activity ranges from 9.1–18.2 mm. *B. pilosa* showed maximum antibacterial activity against *E. coli* with 18.2 mm (10 mg/mL) inhibition zone as compared to standard Ampicillin (30 μg/mL). The extract showed significant antimicrobial inhibition of *S. aureus*, *M. luteus* and *P. aeruginosa* with 15.66, 14.66 and 14 mm at the concentration of 10 mg/mL and was less active against *C. albicans* with 9.1 mm inhibition at the same concentration. However, the extract showed moderate activity at higher concentrations.

**Table 2:** Antimicrobial activity of methanolic extract of *Bidens pilosa* leaves using agar well diffusion method

Test Organisms	Diameter of zone of inhibition (in mm)		ANOVA
	Methanolic extract (Zone of inhibition ± SE)	Ampicillin (30 μg/mL) (Zone of inhibition ± SE)	
*P. aeruginosa*	14.00 ± 0.57	15 ± 0.33	*P* < 0.05
*C. albicans*	9.1 ± 0.33	30 ± 0.25	*P* < 0.05
*E. coli*	18.2 ± 0.35^a^	15 ± 0.10	*P* < 0.05
*S. aureus*	15.66 ± 0.25	15 ± 0.00	*P* < 0.05
*B. subtilis*	3.2 ± 0.25	10 ± 0.33	*P* < 0.05
*M. luteus*	14.66 ± 0.17	15 ± 0.00	*P* < 0.05

^a^Values indicate significant activity against the pathogen

#### Antimicrobial assay using broth micro dilution method

The minimum inhibitory concentrations (MICs) against selected pathogens are represented in Table 3. The extract of *B. pilosa* showed significant activity against selected bacterial pathogens with MIC ranging from 80 to 870 μg/ml. The extract showed maximum activity against *E. coli* (80 μg/mL) followed by *S. aureus* (110 μg/mL) and *P. aeruginosa* (220 μg/mL) [Table 3]. The MIC of extract showed significant effect against pathogenic bacterial strains that means the plant extract has a potential to develop antimicrobial agent.

**Table 3:** Minimum Inhibitory Concentration (MIC) of methanolic extract of *Bidens pilosa* leaves

Test Organisms	MIC values (in μg/mL)			ANOVA
	Methanolic extract (MIC ± SE)	IC_50_ value	Ampicillin (MIC ± SE)	
P. Aeruginosa	220 ± 0.17	250.52	110 ± 0.05	*P* < 0.05
C. Albicans	870 ± 0.25	640.04	210 ± 0.30	*P* < 0.05
E. Coli	80 ± 0.05^a^	110.67^a^	60 ± 0.05	*P* < 0.05
S. Aureus	110 ± 0.17	150.71	82 ± 0.25	*P* < 0.05
B. Subtilis	380 ± 0.27	520.83	230 ± 0.15	*P* < 0.05
M. Luteus	250 ± 0.15	290.11	320 ± 0.05	*P* < 0.05

^a^Values indicate significant activity against the pathogen

### Cytotoxicity assay

**Fig. 3:**
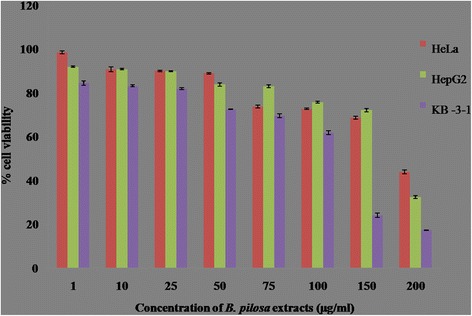
Effect of methanol leaves extract of *B. pilosa* on tested cancer cell lines. Bar represents the means ± SD of triplicate experiments

MTT [3-(4, 5-dimethylythiazol-2-yl)-2, 5-diphenyl-2H- tetrazolium hydrobromide] assay was employed to evaluate the cytotoxicity activity against three cancer cell lines: human epithelial carcinoma (HeLa), human hepato carcinoma (HepG2) and human epidermoid carcinoma (KB-3-1). The IC_50_ value was determined as compared to that of untreated cells and percentage viability curve was plotted against the extract concentration. Microscopic and colorimetric measurements were done after 24 h of treatment with the tested extract. The extract showed significant inhibitory effect against tumour cell growth with varying efficiency. Among the screened cell lines, plant extract showed highest activity against KB-3-1cell lines with IC_50_ values of 99.56 μg/mL (Fig. 3). The IC_50_ values for the inhibition of HepG2 and HeLa cells were found to be 210.8 μg/mL and 179.3 μg/mL respectively. Figure 3 explained that decrease in cell viability which indicated apoptosis induced by methanolic extract of *B. pilosa*. The results indicated that the leaves of *B. pilosa* might contain some anticancerous compounds.

### DNA fragmentation assay

**Fig. 4:**
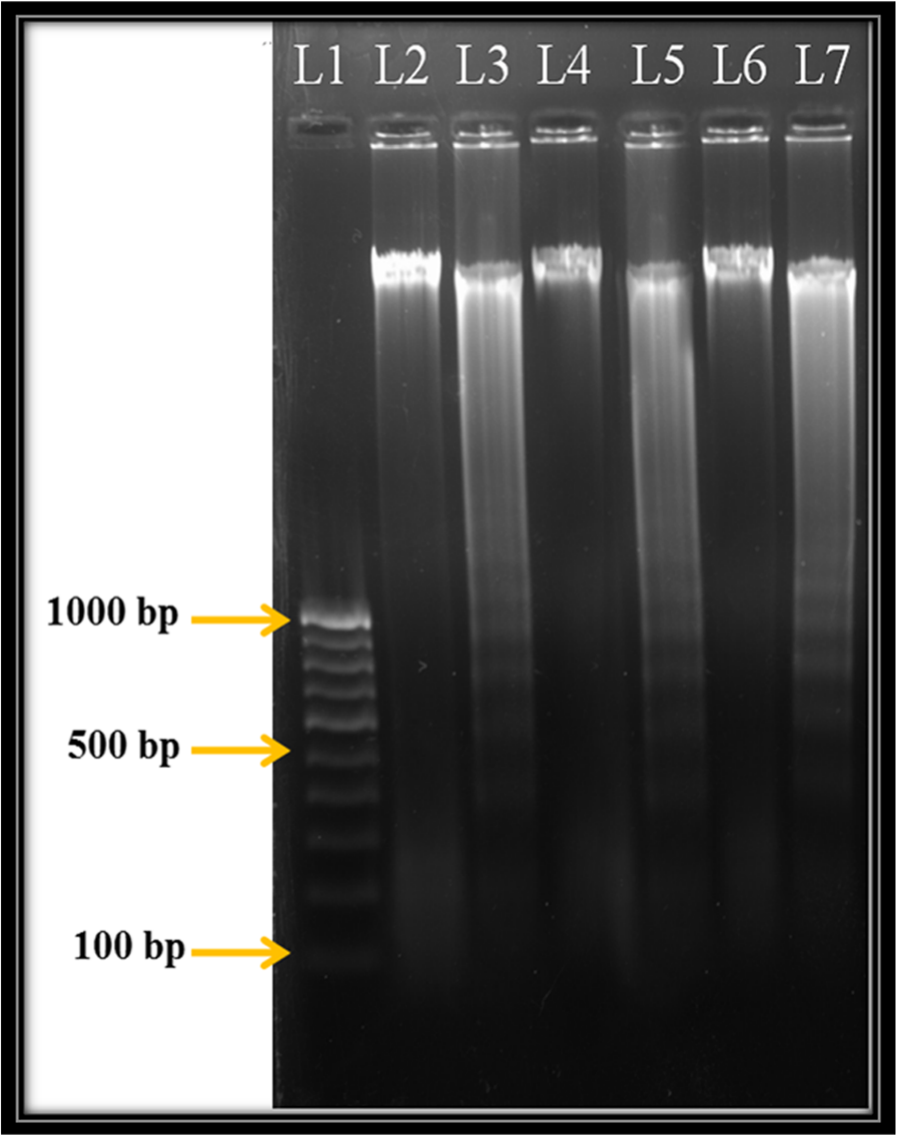
The methanolic extract of *B. pilosa* induced DNA fragmentation. L1–100 bp DNA Ladder; L2- Untreated KB-3 cells DNA; L3- DNA KB-3 cells treated with extract; L4- Untreated HeLa cells DNA; L5- DNA HeLa cells treated with extract; L6- Untreated HepG2 cells DNA; L7- DNA HepG-2 cells treated with extract

DNA fragmentation assay was carried out to understand the possible mechanism of cell death on selected cancer cell lines by the methanolic extract of *B. pilosa.* All the cells were grown and were treated by the IC_50_ concentration of the extract for 72 h. Further, DNA was extracted from the treated cells using 2.0% agarose gel electrophoresis. A typical ladder like pattern was observed which shows the internucleosomal fragmentation. The findings suggested that the methanolic leaf extract of *B. pilosa* is a potent inducer of apoptosis in HeLa, HepG2, and KB-3 cells (Fig. 4).

### Detection and quantification of phenolic compounds by UHPLC-QqQ_LIT_-MS/MS

#### Analytical method validation

Determination and quantitative analysis of bioactive compounds was performed using UHPLC-MRM method as described earlier Chandra et al. [39].

### Linearity, limits of detection (LOD) and quantification (LOQ)

Calibration curves of standard compounds were established using different concentrations of reference analytes. LOD and LOQ were determined using diluted standard compounds when the signal-to-noise rations of reference analytes were about 3 and 10, respectively. The obtained results are listed in Table 4. The calculations for calibrations curves and correlation coefficients (r^2^) were from 0.9996 to 1.0000 within test ranges. LOD and LOQ of reference analytes was 0.01 to 0.20 ng/ml and 0.03 to 0.61 ng/ml respectively.

**Table 4:** Method validation parameters for five reference analytes

Parameters	Analytes				
	Catechin	Kaempferol	Ferulic acid	Gallic acid	Paclitaxel
Regression equation	6.01*×* + 0.33	5.53*×* + 0.31	40.02*×* + 0.05	41.44*×* + 0.02	306× + 1.92
Correlation coefficient (r^2^)	0.9998	0.9999	0.9995	1.0000	0.9996
Linearity range (ng/mL)	1–250	1–250	0.5–100	0.1–100	5–500
LOD (ng/mL)	0.14	0.20	0.03	0.01	0.02
LOQ (ng/mL)	0.43	0.61	0.09	0.03	0.07
Precision RSD % (Intra-day, *n* = 6)	0.34	1.02	0.62	0.25	0.61
Precision RSD % (Inter-day, n = 6)	1.01	1.22	1.11	0.94	1.21
Stability RSD % (*n* = 5)	1.83	2.55	1.88	1.92	1.80
Recovery (*n* = 3) Mean	105.19	97.50	94.87	95.50	98.86
RSD %	0.76	1.02	1.13	1.82	0.96

### Precision, stability and recovery

Relative standard deviation (RSD) was used to measure precision and intra-day and inter-day variations were evaluated by using six replicates and repeating the experiments for 3 days. The intra-day and inter-day precision was found to be less than 1.21%. Stability was also measured by replicating the injections at 0, 2, 4, 8, 12 and 24 h. The percentage of RSD of five standard analytes was found to be 2.55. The method developed for evaluation of bioactive compounds from *B. pilosa* leaves extract has good accuracy, with recovery ranges from 94.87% to 105.19% for all analytes (Table 4).

### Quantitative analysis

**Fig. 5:**
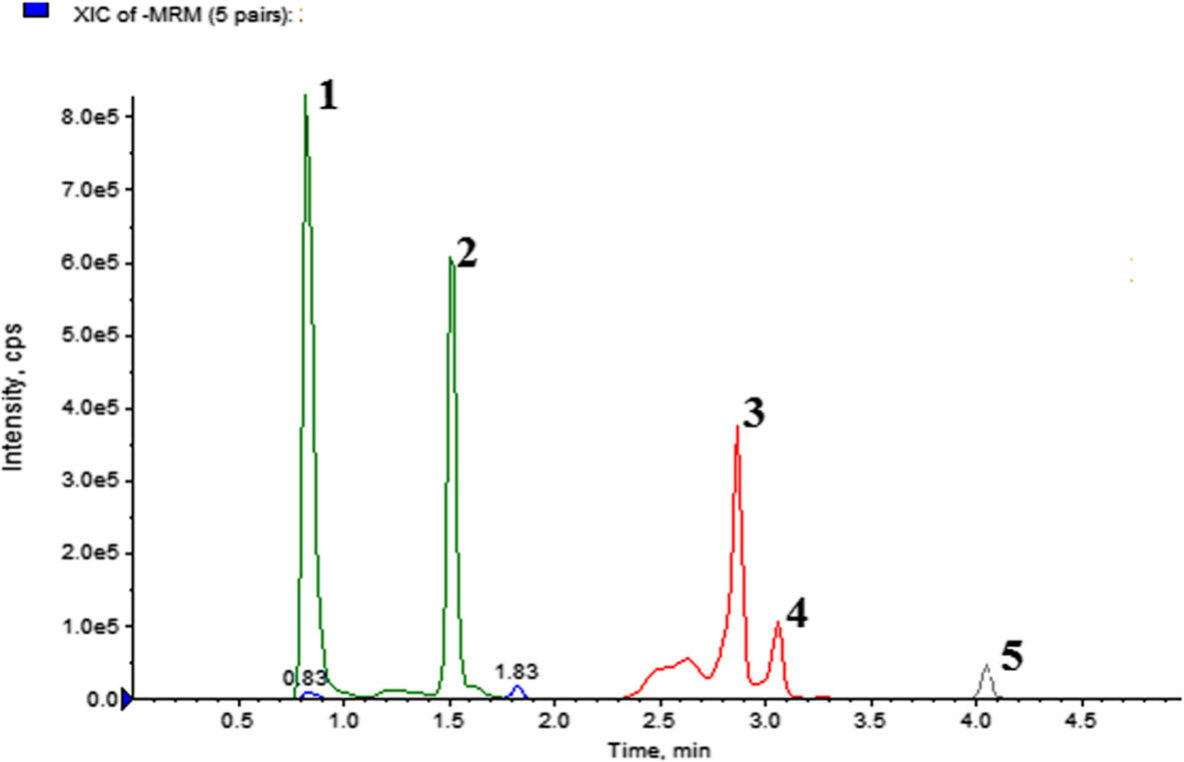
MRM extracted chromatogram of standards bioactive compounds mixture obtained by UPLC-ESI–MS/MS in negative mode

**Fig. 6:**
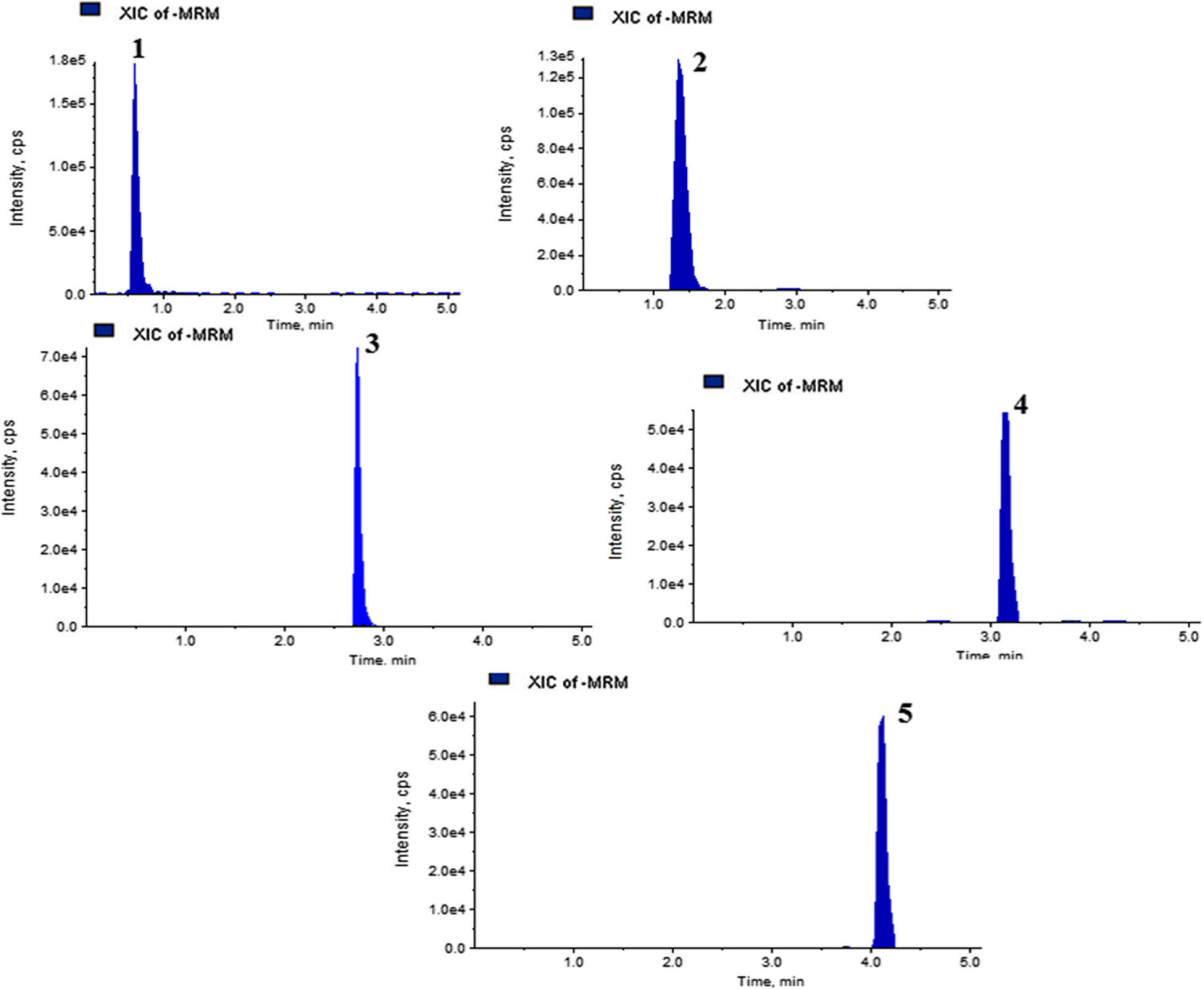
Extracted ion chromatograms (XICs) of five pairs of standard bioactive compounds obtained by UPLC-ESI–MS/MS

**Fig. 7:**
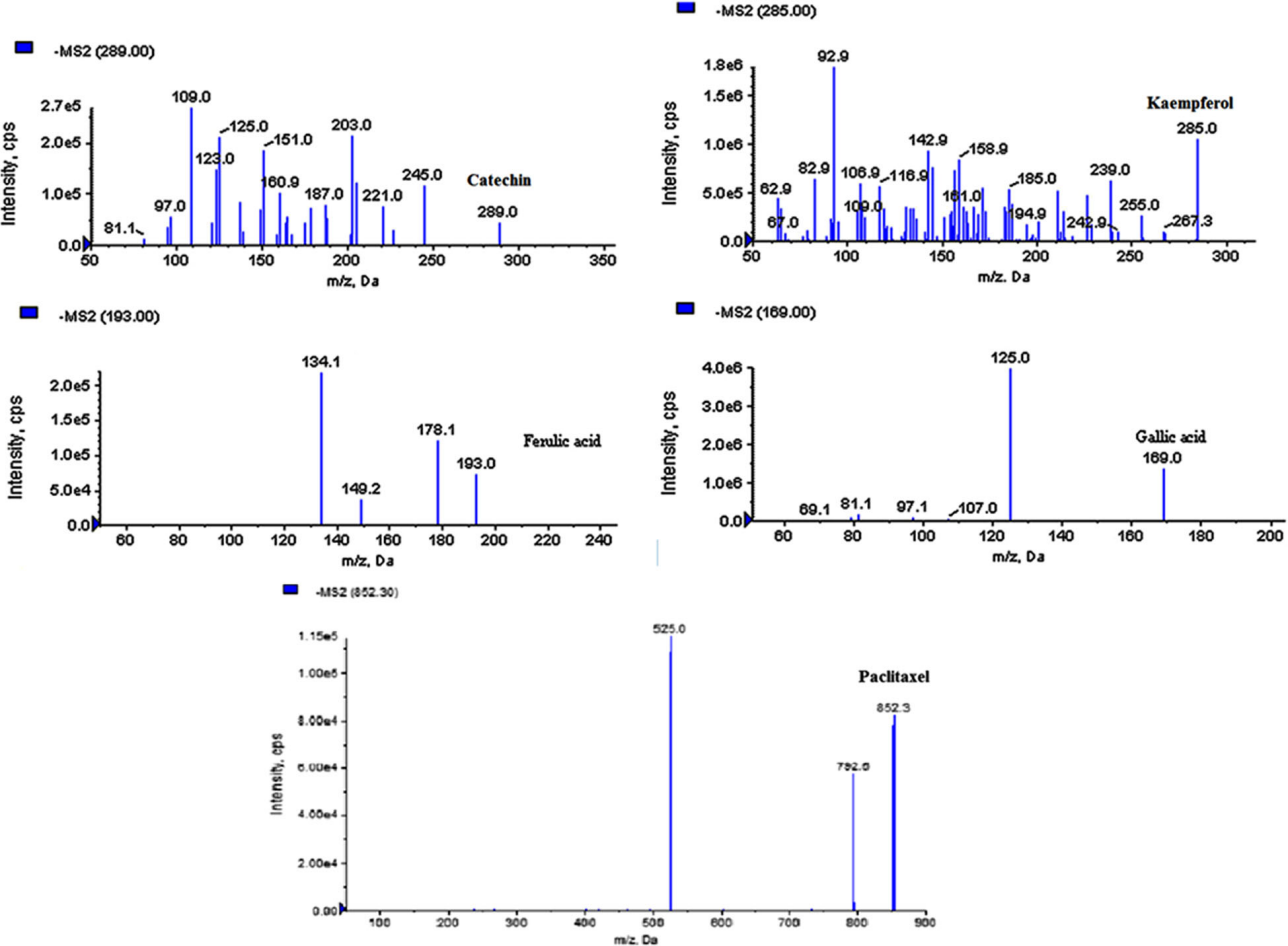
MS/MS spectra of target phenolic and anticancerous compounds

In this study, the UHPLC-QqQ_LIT_-MS/MS method was applied to five quantitative reference compounds. Quantitative results are listed in Table 5. Gallic acid (33.3 mg/g) was present at the highest amounts, while ferulic acid (0.58 mg/g) was lowest in *B. pilosa*. The findings of the study prove the existence of variations among the tested reference analytes in *B. pilosa*. The MRM, extracted ion chromatogram and MS/MS spectra of five mixed standards are shown in Figs. 5, 6 and 7.

**Table 5:** Content (mg/g) of five bioactive compounds detected in *B. pilosa*

Analyte name	Analytes concentration (mg/G)
Catechin	16.0
Kaempferol	32.87
Ferulic acid	0.58
Gallic acid	33.3
Paclitaxel	15.0

### Analysis of volatile compounds by gas chromatography-mass spectroscopy (GC-MS)

**Fig. 8:**
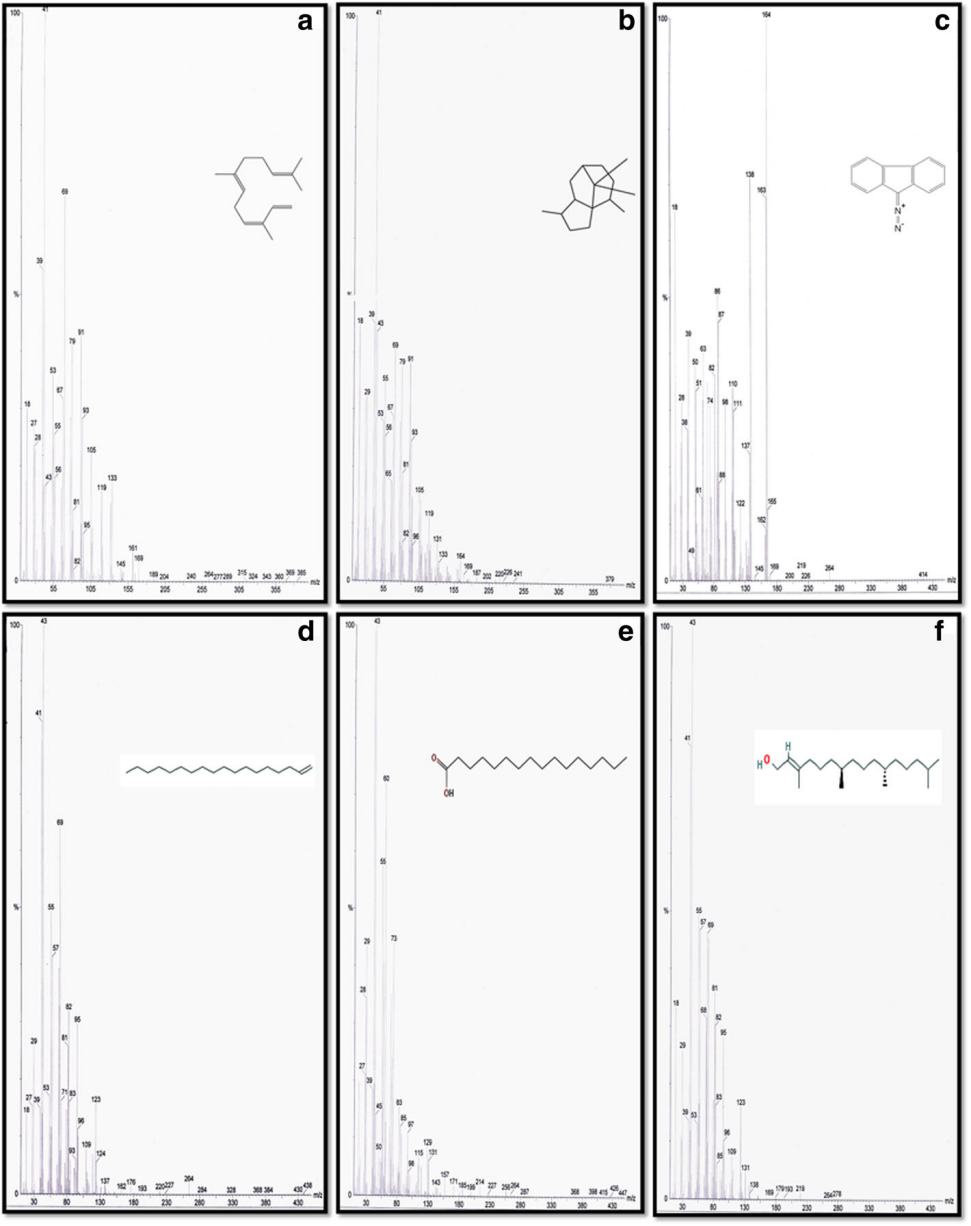
GC-MS Chromatogram detected six volatile compounds from methanolic extract of *Bidens pilosa* plant compared with NIST library. **a** 1,3,6,10-Dodecatetraene, 3,7,11-trimethyl-(Z,E); (**b**) 1H-3A, 7-Methanozulene, Octahydro-1,4,9,9-tetramethyl; (**c**) 9H–Fluorene, 9-Diazo; (**d**) 1-Octadecyne; (**e**) N-Hexadecanoic acid and (**f**) 3,7,11,15-Tetramethyl-2-Hexadecen-1-ol

GC-MS analysis of compounds was performed in methanolic leaf extract of *B. pilosa*, shown in Table 6. The identification of volatile compounds is based on the peak area, retention time, percentage of area, molecular weight and molecular formula. Several compounds were detected in the methanolic leaves extract of *B. pilosa* including 1,3,6,10-Dodecatetraene, 3,7,11-trimethyl-(Z,E); 1H-3A, 7-Methanozulene, Octahydro-1,4,9,9-tetramethyl; 9H–Fluorene, 9-Diazo; 1-Octadecyne; N-Hexadecanoic acid and 3,7,11,15-Tetramethyl-2-Hexadecen-1-ol. The spectrum profile of GC-MS analysis showing six components individual fragmentation pattern with retention time 14.05, 16.18, 17.80, 18.90, 20.16 and 21.62 is demonstrated in Fig. 8. The highest peak area (%) of 57.82 was found in 3,7,11,15-Tetramethyl-2-Hexadecen-1-ol with retention-time 21.62 and the lowest peak area (%) of 3.96 was detected in 1,3,6,10-Dodecatetraene, 3,7,11-trimethyl-(Z,E) with retention-time 14.05 (Table 6).

**Table 6:** Volatile compounds identified in the methanolic leaf extract of *Bidens pilosa* by GC-MS

Sl. No.	Name of the Compound	RT	Peak Area	Area (%)	Height	Molecular Weight	Nature of compound
1	1,3,6,10-Dodecatetraene, 3,7,11-trimethyl-(Z,E)	14.053	683,540.1	3.96	18,457,168	204.3511	
2	1H-3A, 7-Methanozulene, Octahydro-1,4,9,9-tetramethyl	16.184	1,116,642.1	6.46	30,036,296	206.3669	
3	9H–Fluorene, 9-Diazo	17.8	3,135,375.3	18.14	82,788,704	192.22	Alkene
4	1-Octadecyne	18.9	1,195,249.8	6.92	33,715,172	252.486	Alkene
5	N-Hexadecanoic acid	20.16	1,158,552.5	6.70	27,304,302	256.4241	Fatty acid
6	3,7,11,15-Tetramethyl-2-Hexadecen-1-ol	21.626	9,990,602.0	57.82	230,113,984	296.539	Fatty acid

### Mosquitocidal bioassay

#### Mortality

Mortality rate (MR) of the third instar larva of *C. quinquefasciatus* treated with methanolic extracts of *B. pilosa* is illustrated in Table 7. The MR of *B. pilosa* was highest at 1000 ppm concentration at different time intervals (*P* < 0.05) at 24 and 48 h of exposure (Table 8). We have found that the highest larvicidal activity (100%) was detected in methanolic extract of *B. pilosa* after 12 h. At higher concentrations, the larvae moved for some time and then died.

**Table 7:** Time dependent mortality check of larvicidal activity of crude methanolic extract of *B. pilosa* till 48 h at different concentrations

Plant extract	Concentration in PPM	% Mortality ± SE (Time in h)									
		1	3	6	12	18	24	30	36	42	48
Methanolic leaf extract of *B. pilosa*	50	0	0	0	4.1 ± 0.25	11.6 ± 0.12	21.5 ± 0.25	37.2 ± 0.17	46.3 ± 0.10	54.7 ± 0.20	67.1 ± 0.20
	100	0	0	6.3 ± 0.10	11.6 ± 0.25	22.4 ± 0.10	38.2 ± 0.10	47.6 ± 0.17	59.2 ± 0.10	67.4 ± 0.25	83.4 ± 0.27
	200	0	0	14.7 ± 0.17	29.4 ± 0.12	42.3 ± 0.27	54.6 ± 0.25	67.0 ± 0.10	76.2 ± 0.05	85.4 ± 0.15	97.1 ± 0.10
	300	0	11.2 ± 0.10	19.8 ± 0.05	27.8 ± 0.15	39.4 ± 0.25	59.4 ± 0.15	74.4 ± 0.17	86.5 ± 0.25	100.0 ± 0.00	–
	400	16.2 ± 0.17	27.8 ± 0.25	41.6 ± 0.05	58.3 ± 0.15	74.8 ± 0.10	89.7 ± 0.15	100.0 ± 0.00	–	–	–
	500	24.2 ± 0.10	47.1 ± 0.05	69.8 ± 0.15	90.8 ± 0.17	100.0 ± 0.00	–	–	–	–	–
	1000	24.1 ± 0.27	42.8 ± 0.10	67.6 ± 0.25	100 ± 0.00	–	–	–	–	–	–
	Control	0	0	0	0	0	0	0	0	0	0

**Table 8:** Log probit and regression analysis of third larval instars of *C. quinquefasciatus* in different concentrations of methanolic extract of *B. pilosa* for 24 h and 48 h

Plant extract	Time	Chi Square	LC_50_ (ppm)	95% confidence limits		df	*R* ^2^ Value	Slope ± SE	Intercept ± SE	*F* value	*P* value
				Lower limit	Upper limit						
Methanolic leaf extract of *B. pilosa*	24 h	0.00	148.7	89.3	247.7	4	0.96	0.168 ± 0.015	17.02 ± 4.69	118.7	0.0004
	48 h	0.667	101.7	94.4	109.5	4	0.99	0.063 ± 0.022	74.87 ± 6.69	8.27	0.045

### Dose-response (LC_50_) and time-response (LT_50_) larvicidal bioassay

Table 8 described the lethal concentration (LC_50_) values of the larvicidal assay after 24 and 48 h of *B. pilosa*. The highest larvicidal activity was found in methanolic extract of *B. pilosa* (LC_50=_ 148.7) after 24 h and (LC_50=_ 101.7) after 48 h. Chi-square value was highly significant at *P* < 0.045 to 0.0004 levels in *B. pilosa* plant extract. The result of one way ANOVA of methanolic extract of *B. pilosa* at different concentrations (50–1000 ppm) and at different time intervals (24 and 48 h) also exhibited significant difference in larval mortality (*P* < 0.0004). Higher slope value (0.168 ± 0.015 at 24 h; 0.063 ± 0.022 at 48 h) and lower and upper limits at 95% confident level of LC_50_ (89.3–247.7 ppm at 24 h; 94.4–109.5 ppm at 48 h) were observed for methanolic extract of *B. pilosa*. The regression analysis showed a positive correlation among the mortality rate (Y) and the concentration of exposure (X) having a regression coefficient (R^2^) of 0.96 and 0.99 respectively. Time response larvicidal bioassay was carried out in methanolic extract of *B. pilosa* at different concentrations (50–1000 ppm) for 48 h against *C. quinquefaciatus*. Methanolic extract of *B. pilosa* has taken minimum lethal time (LT_50_ = 6 h) to kill 50% of *C. quinquefasciatus* at 500 ppm (Table 9). Stastical analysis showed a positive correlation between the LT_50_ values and mortality rate was found. Significant Chi-square value (at *P* < 0.008 to 0.0001 level), higher slope value (2.212 ± 0.101 at 300 ppm) and lower and upper limits at 95% confident level of LT_50_ (4.28 at 500 ppm & 29.98 at 50 ppm) were also observed in methanolic extract of *B. pilosa*.

**Table 9:** Log probit and regression analysis of time dependent larvicidal efficacy of methanolic extract of *B. pilosa* at different concentrations against third instar larvae of *C. quinquefasciatus*

Plant name	Concentration	Chi Square	LT_50_ (h)	95% confidence limits		df	*R* ^2^_Value_	Slope ± SE	Intercept ± SE	*F* value	*P* value
				Lower limit	Upper limit						
Methanolic extract of *Bidens pilosa*	50	2.8	28.32	26.74	29.98	8	0.96	1.488 ± 0.097	−8.47 ± 2.64	232.9	0.0001
	100	0.8	25.36	23.09	27.86	8	0.99	1.788 ± 0.057	−5.734 ± 1.566	959.1	0.0001
	200	0.8	19.17	16.74	21.95	8	0.98	2.087 ± 0.083	0.76 ± 2.25	628.8	0.0001
	300	0.8	18.85	15.67	22.67	8	0.98	2.212 ± 0.101	3.196 ± 2.76	471.3	0.0001
	400	5.4	10.17	8.34	12.4	4	0.86	1.815 ± 0.258	30.91 ± 7.0	49.45	0.0001
	500	10.0	4.692	4.28	5.14	7	0.99	1.262 ± 0.367	55.43 ± 9.95	11.8	0.008

## Discussion

Phenolics are one of the vital groups of secondary metabolites present in plants. Rose and Kasum, [40] suggested that the phenolic compounds helps in the maintenance of human health by protecting against various diseases. Moreover, flavonoids are a group of phenolics which have broad spectrum antioxidant properties. In the present study, the TPC was estimated to be 72 μg of GAE/mg of DW significantly high than the reported by Lee et al. [41] from *B. pilosa* (38.1 mg of GAE/g of DW). The total flavonoids content was found to be 123.3 μg Quercetin/mg of DW. The findings were in support of Lee et al. [38] who demonstrated the TFC as 235.06 mg Quercetin/g of DW. The higher amount of phenolic and flavonoids production showed better antioxidant capacity of the tested extract [42]. Cortés-Rojas et al. [43] suggested that the leaves and flowers of *B. pilosa* have highest TPC and TFC contents as compared to other parts. The main role of flavonoids in the plants is to protect plants from sun radiation and scavenge free radicals. Hence, it is quite expected that the plant parts exposed to sunlight are high in the TFC [44].

Free radicals are well known to play quiet effective role in pathological symptoms [44]. Antioxidant helps us from various diseases by protecting against free radicals either by scavenging the reactive oxygen species or protecting the cells by antioxidant defence mechanisms [45]. *B. pilosa* methanolic extract was tested for free radical scavenging ability using DPPH and ABTS method. In our study, we found that DPPH IC_50_ value of 80.45 μg/ml in methanolic extract of *B. pilosa*. Adedapo et al. [46] reported that DPPH IC_50_ value of 94.2 mg/mL which was higher than our reported value. Deba et al. [1] reported that antioxidant activity of essential oils from *B. pilosa* and showed that leaves and flowers essential oil having DDPH IC_50_ value of 47 and 50 μg/ml respectively which further proved that leaves has the highest antioxidant potential as compared to other parts of the selected plants. ABTS, A more appropriate decolorization technique assay in which the radicals are generated directly in a stable form prior to reaction with putative antioxidants [26]. In our study, ABTS IC_50_ value of 171.6 μg/ml which is higher than the previously reported by Adedapo et al. [47] who showed IC_50_ value of ABTS as 0.75 mg/mL.

The antimicrobial activity showed that *B. pilosa* have significant antimicrobial potential against four human bacterial pathogen (*S. aureus, P. aeruginosa, M. luteus* and *E. coli*) and yeast *C. albicans* which are the most common cause of different food borne diseases. In this study, methanolic extract of *B. pilosa* exhibited significant inhibitory effect against gram-negative bacteria (18.1 mm diameter zone of inhibition) than the gram positive bacteria (14.6 mm diameter zone of inhibition) which is compared to standard known antibiotics ampicillin (50 μg/disc). The highest zone of inhibition was found against *E. coli* (18.2 mm). The findings of zone of inhibition was slightly higher than a study reported by Falowo et al. [47] who stated that methanolic extract of *B. pilosa* showed zone of inhibition against *E. coli* (16.0 mm).

We found that *B. pilosa* leaves extract exhibited significant antibacterial activity against *S. aureus* (15.6 mm). This result was similarly reported by Ashafa and Afolayan, [48] who demonstrated that methanolic extract of *B. pilosa* have suppressed the growth of Gram positive bacteria *S. aereus* (5.0 mm). According to some previous researchers, methanolic extract of *B. pilosa* was inactive against *P. aeruginosa* and *S. aureus* [46, 47]. As these bacteria are having resistant capacity against the extracts could be characterized to their cell wall which has been mentioned to inhibit the penetration of the plant extract [49, 50].

The minimum inhibitory concentration (MICs) of methanolic extracts of *B. pilosa* against selected bacterial pathogens is represented in Table 3. The methanolic extracts of *B. pilosa* inhibited bacterial and yeast pathogen with MIC ranging from 80 to 380 μg/mL. *B. pilosa* showed highest activity against *E. coli* (80 μg/mL) followed by *S. aureus* (110 μg/mL) and *P. aeruginosa* (220 μg/mL). Previous reports also showed that the methanolic leaves extract was more active which indicates that the methanolic leaves extract has the potential antimicrobials [48]. The *B. pilosa* extract showed significant inhibitory activity against bacterial and yeast pathogen which suggest as an exploitable source for the discovery of antimicrobial agents [30, 51].

Previous reports have stated that isolated new compounds from *B. pilosa* have anticancer activities against various types of cancer. According to Kviecinski and Felipe, [20], different crude extract like chloroform, ethyl acetate and methanol fractions of *B. pilosa* possess anti-tumor activity which has assessed using brine shrimp, hemolytic, MTT, and neutral red uptake (NRU) assays. In present study, the methanolic extract of *B. pilosa* inhibited the growth of three cancer cell lines KB-3-1, HepG2 and HeLa with IC_50_ values of 99.56 μg/mL, 210.8 μg/mL and 179.3 μg/mL respectively. Percentage of inhibition was found significantly high than the previous studies reported by Sundararajan et al. [9] and Wu et al. [52] who stated that the methanol extract of *B. pilosa* showed anticancer activity against HeLa, HepG2 and KB cells with IC_50_ values of 965.2 μg/mL, 119.55 μg/mL and 586.2 μg/mL respectively. Steenkamp and Gouws, [53] reported that several members of *Asteraceae* family such as *B. pilosa* showed cytotoxic activity on some tumor cell lines. Furthermore, Kumari et al. [10] reported that the isolated compound phenyl-1, 3, 5-heptatriene from *B. pilosa* has antiproliferating effect against human oral, liver, colon, and breast cancer cell lines with IC_50_ values of 8.0 ± 0.01, 0.49 ± 0.45, 0.7 ± 0.01and 10 ± 0.01 μg/mL respectively. Further, DNA fragmentation was observed in HeLa, HepG2, and KB-3 cells treated with *B. pilosa* extract, thereby indicating the onset of apoptotic cell death. Thus, the results obtained in this study suggest that the methanolic extract of *B. pilosa* might have an apoptosis-inducing property, isolated from the leaves of *B. pilosa* can act as potential anticancer agents in cancer chemotherapy.

A few phenolic compounds like gallic acid, Kaempferol, Catechin, Paclitaxel and Ferulic acid was detected for the first time from methanolic extract of *B. pilosa* plant. Kaempferol, phenolic compound was also reported first time from *B. pilosa* which is used for the treatment of various types of cancers [54, 55]. Ferulic acid was detected in less quantity (0.58 mg/G). This compound was similarly reported by Muchuweti et al. [56] who has detected from 50% aqueous methanol of *B. pilosa* using HPLC system. Paclitaxel, brand name taxol is a chemotherapy medication which was reported first time from *B. pilosa.* This compound was isolated first time from the bark of the Pacific yew, *Taxus brevifolia* and its given name “taxol” [57].

GC-MS analysis of the methanolic extract of *B. pilosa* showed the presence of six volatile compounds i.e. 1,3,6,10-Dodecatetraene, 3,7,11-trimethyl-(Z,E); 1H-3A, 7-Methanozulene, Octahydro-1,4,9,9-tetramethyl; 9H–Fluorene, 9-Diazo; 1-Octadecyne; N-Hexadecanoic acid and 3,7,11,15-Tetramethyl-2-Hexadecen-1-ol. These compounds are responsible for numerous pharmacological actions like antimicrobial activities useful in a treatment of variety of diseases and anticancer activities against various cancers [58, 59]. Recently, Kale, [59] reported that two volatile compound namely N-Hexadecanoic acid and 3,7,11,15-Tetramethyl-2-Hexadecen-1-ol from ethanolic leaf extract of *Adiantum capillus-veneris* L which has similarly reported in our study from methanolic extract of *B. pilosa* plant. To best our knowledge, this is first time reported six compound 1,3,6,10-Dodecatetraene, 3,7,11-trimethyl-(Z,E); 1H-3A, 7-Methanozulene, Octahydro-1,4,9,9-tetramethyl; 9H–Fluorene, 9-Diazo; 1-Octadecyne; N-Hexadecanoic acid and 3,7,11,15-Tetramethyl-2-Hexadecen-1-ol from methanolic extract of *B. pilosa.*

Larvae are mainly killing by using different synthetic chemicals like – organochlorine (DDT), organophosphates (malathion, temephos and fenthion), synthetic pyrethroids (deltamethrin), insect growth regulators (diflubenzuron and methoprene) etc. A high amount of DDT and Malathion resistance was used in *C. quinquefasciatus* last several years in Northeast India. The use of DDT is stopped in several places of India due to development of resistance in vector populations. Though, this chemicals are still used for control of Kala-azar vector and malaria vectors of different parts of North-eastern India especially Mizoram [60]. Since insecticide resistance threatens to contribute towards the reintroduction of vector borne diseases in many parts of the world, efforts have been focused on finding an alternative form of mosquito control. Therefore, several compounds of plant have been reported as insecticides-larvicides which are very essential to improve their formulations with enhanced activity. So, this improved product may be useful to control insecticides and mosquito. Previous researchers reported that different plant families – Asteraceae, Solanaceae, Euphorbiaceae, Leguminoceae, Cladophoraceae, Labiatae, Meliaceae, Solanaceae, Umbelliferae, Compositae, Myrtaceae, Lauraceae, Lamiaceae, Apiaceae, Cupressaceae, Poaceae, Zingiberaceae, Piperaceae, Aristolochiaceae, Caesalpinaceae, Chenopodiaceae, Oocystaceae, Fabaceae and Rutaceae showed larvicidal and insecticidal activity against different species of mosquitoes [61–64]. The crude methanolic extract of *B. pilosa* showed larvicidal effect against third instar larva of *C. quinquefasciatus*. The methanolic extract of *B. pilosa* exhibited 100% mortality rate after 12 h of incubation at the concentration of 1000 ppm. Similarly, Macêdo et al. [65] checked that ethanolic extract of *Bidens pilosa* showed larvicidal effect against fourth instar larva of *Aedes fluviatilis* who stated that 12.2% of mortality at 100 mg/L concentration. To best our knowledge, this is first time reported that methanolic extract of *B. pilosa* exhibited larvicidal activity against third instar larva of *C. quinquefasciatus.*

## Conclusions

The overall findings of our study provide evidence for the bioactive potential of methanolic leaves extract of *B. pilosa* and the ecological significance of human well being. The results obtained bring up supporting data for future investigation of the studied plant which could lead to their use in cancer, oxidative stress and antimicrobial therapy.

## Abbreviations

ABTS^+^: 2,2- Azinobis-3-ethylbenzothiazoline-6-sulphonic acid disodium salt
AlCl3: Aluminium chloride
ANOVA: Analysis of variance
CFU: Colony forming unit
DMSO: Dimethyl sulfoxide
DPPH: 2,2-diphenyl-1-picrylhydrazyl
GC-MS: Gas chromatography-mass spectrometry
HeLa: Human epithelial carcinoma
HepG2: Human hepato carcinoma
IC_50_: The Inhibitory concentration required for 50% scavenging of DPPH and ABTS radicals in a specific time
KB-3-1: Human epidermoid carcinoma
LC_50_: Lethal concentration required to kill 50% of mosquito larvae
LT_50_: Lethal time till half the mosquito larvae dies
MIC: Minimum inhibitory concentrations
MRM: Multiple Reaction Monitoring
MTCC: The Microbial Type Culture Collection and Gene Bank
MTT: 3-(4, 5-dimethylythiazol-2-yl)-2, 5-diphenyl-2H- tetrazolium hydrobromide
UHPLC-QqQ_LIT_-MS/MS: Ultra high performance liquid chromatography-hybrid linear ion trap triple quadrupole mass spectrometry
